# Glutathione Depletion Disrupts Redox Homeostasis in an Anoxia-Tolerant Invertebrate

**DOI:** 10.3390/antiox12061197

**Published:** 2023-05-31

**Authors:** Marlize Ferreira-Cravo, Daniel C. Moreira, Marcelo Hermes-Lima

**Affiliations:** 1 Department of Cell Biology, University of Brasilia, Brasilia 70910-900, Brazil; 2 Federal University of Mato Grosso do Sul, Campo Grande 79070-900, Brazil; 3 Research Center in Morphology and Applied Immunology, Faculty of Medicine, University of Brasilia, Brasilia 70910-900, Brazil

**Keywords:** hypoxia, ischemia, preparation for oxidative stress, reoxygenation, reperfusion

## Abstract

The upregulation of endogenous antioxidants is a widespread phenomenon in animals that tolerate hypoxia/anoxia for extended periods. The identity of the mobilized antioxidant is often context-dependent and differs among species, tissues, and stresses. Thus, the contribution of individual antioxidants to the adaptation to oxygen deprivation remains elusive. This study investigated the role of glutathione (GSH) in the control of redox homeostasis under the stress of anoxia and reoxygenation in *Helix aspersa*, an animal model of anoxia tolerance. To do so, the total GSH (tGSH) pool was depleted with l-buthionine-(S, R)-sulfoximine (BSO) before exposing snails to anoxia for 6 h. Then, the concentration of GSH, glutathione disulfide (GSSG), and oxidative stress markers (TBARS and protein carbonyl) and the activity of antioxidant enzymes (catalase, glutathione peroxidase, glutathione transferase, glutathione reductase, and glucose 6-phosphate dehydrogenase) were measured in foot muscle and hepatopancreas. BSO alone induced tGSH depletion by 59–75%, but no other changes happened in other variables, except for foot GSSG. Anoxia elicited a 110–114% increase in glutathione peroxidase in the foot; no other changes occurred during anoxia. However, GSH depletion before anoxia increased the GSSG/tGSH ratio by 84–90% in both tissues, which returned to baseline levels during reoxygenation. Our findings indicate that glutathione is required to withstand the oxidative challenge induced by hypoxia and reoxygenation in land snails.

## 1. Introduction

Many animal species tolerate drastic changes in the natural environment, which may become unsuitable for development, growth, and reproduction within varying ranges of time, from hours to weeks. Such harsh conditions include wide fluctuations in temperature, salinity, air humidity, oxygen availability, food and/or water supply, and UV incidence [1,2,3]. The biochemical adaptation program activated under such conditions includes the activation of endogenous antioxidants to cope with large fluctuations in the generation of reactive oxygen and nitrogen species (RONS) that are expected to accompany these natural, but potentially stressful, events. The upregulation of antioxidant systems under environmental stress was coined “Preparation for Oxidative Stress” (POS) [2,4,5], which has been described to occur in over 80 animal species from 8 phyla, under laboratory simulations of harsh environmental conditions [6,7]. More recently, the POS phenotype has also been observed to happen naturally in the field for a few animal taxa, including mollusks [8,9,10,11].

Since its conception, the mechanisms leading to POS remained elusive for a long time [12]. Only a few year ago, we proposed that the POS phenotype (i.e., the activation of endogenous antioxidants) is the result of RONS-induced activation of redox-sensitive transcriptional factors, prompting the increase in the expression of enzymes involved in the management of oxidative stress [13]. Experimental evidence suggests that RONS-mediated activation of kinases may also activate the POS phenotype through a hormetic process [14]. Together, these processes ultimately lead to the increase in the expression and/or activity of enzymes involved in the management of reactive species and oxidative stress [13]. Rather than a global upregulation of all endogenous antioxidants, often the identity of the upregulated antioxidant is context-dependent, depending on tissue, species, and stress [2,15]. For example, glutathione peroxidase and catalase are the most commonly upregulated antioxidants in animals exposed to anoxia [2], highlighting the relevance of catalase- and glutathione-dependent systems in the POS response and adaptation to anoxia.

One challenge in the field lies in demonstrating the relevance of individual antioxidants for the functionality of the POS mechanism and overall fitness of animals, as most studies typically measure the levels of only a few selected antioxidants rather than multiple components of antioxidant systems simultaneously. A few years ago, this issue was addressed by studying the effects of injecting 3-amino-1,2,4-triazole (ATZ), a catalase inhibitor, on redox balance. When land snails (*Helix aspersa*) or Nile tilapia (*Oreochromis niloticus*) were exposed to low or very low oxygen tensions in the presence of pharmacologically inhibited catalase, significant changes in markers of redox metabolism were observed [16,17]. Specifically, animals injected with ATZ showed signs of oxidative stress compared to animals injected with saline. These studies highlighted the role of catalase and H_2_O_2_ in the biochemical mechanism of POS in both species. Here, we aimed to investigate the role of glutathione, another antioxidant, in the biochemical response of *H. aspersa* to anoxia. To achieve this, we depleted glutathione levels by injecting buthionine sulfoximine (BSO), an inhibitor of γ-glutamylcysteine synthetase. The animals were then exposed to anoxia and reoxygenation. Subsequently, the activity of antioxidant enzymes and levels of oxidative stress markers were measured at various time points in the hepatopancreas and skeletal muscle. Animals that evolved under the pressure of periodic events of oxygen deprivation have been widely used as models of hypoxia tolerance, an ability that most mammal cells lack [18,19,20,21,22]. Land snails *Helix* spp. are representative of such species that withstand long periods of complete lack of oxygen [17,23,24,25], being experimental models to study biochemical adaptations associated with anoxia tolerance.

## 2. Materials and Methods

### 2.1. Animals

The study utilized *Helix aspersa* maxima (Müller, 1774) (currently renamed *Cornu aspersum*) as a resilient experimental model for hypoxia. This species can tolerate up to 15 h of anoxia [26]. Snails weighing approximately 15 g were obtained from a commercial supplier located in the state of Sao Paulo, Brazil (Helix Escargots). Upon arrival at the laboratory, the snails were placed in transparent plastic boxes and provided with a balanced diet (prepared as described in Ref. [17]) and unlimited access to water. The animal boxes were replaced daily with clean ones. The laboratory conditions included a photoperiod of 12 h of light and 12 h of darkness, with the temperature maintained at 23 ± 1 °C. The snails were acclimatized to these conditions for at least three weeks before any experimentation took place. The experiments were conducted during March and April.

### 2.2. Glutathione Depletion

The levels of glutathione in *H. aspersa* tissues were pharmacologically depleted by injecting l-buthionine-(S,R)-sulfoximine (BSO), a specific inhibitor of γ-glutamate-cysteine ligase (GCL) activity. This enzyme catalyzes the rate-limiting step of glutathione synthesis [27,28]. Based on previous studies involving invertebrates and fish [29,30,31], snails were injected with a dose of 1 g BSO per 1 kg of body weight. Pilot experiments indicated that this dosage was sufficient to reduce total glutathione levels by 60–70% in the foot muscle and hepatopancreas of snails 72–96 h after injection. A stock solution of BSO was prepared in physiological saline and injected at a volume of 12.5 µL per gram of body weight, excluding the shell mass (considering that the shell accounts for 23% of the total weight, as per [17]).

One group of animals received the BSO injection at the aforementioned dosage (BSO group), while another group received a parallel injection of saline using the same volume-to-weight ratio (saline group). Control animals did not receive any injections (control group). The injection was conducted using a Hamilton syringe through the foot muscle, which allowed the drug to enter the celomic cavity of the snail. The injection was performed using a Hamilton syringe through the foot muscle, entering the celomic cavity of the snail, simulating what would be an intraperitoneal injection in mammals. This method has been previously effective in delivering drugs to the hepatopancreas and skeletal muscle of snails [17].

### 2.3. Anoxia and Reoxygenation

After the injection of either saline or BSO, the snails were maintained under normoxia for 88.5 h before being exposed to anoxia. This synchronization was necessary to align the peak of glutathione depletion with the onset of oxygen stress. The snails were placed in glass containers measuring 20 cm × 19.5 cm × 12.5 cm, with one opening connected to a nitrogen gas cylinder (in) and another opening for gas flow (out). Anoxia was achieved by flushing nitrogen gas for 30 min, after which the containers were sealed and maintained under anoxia for 5.5 h. At the end of the anoxia period, a subset of animals was immediately euthanized, while another subset was allowed to reoxygenate for up to 2 h.

Within each glass container, a group of 5 animals was placed, representing the anoxia, reoxygenation for 15 min, reoxygenation for 30 min, reoxygenation for 1 h, and reoxygenation for 2 h groups, respectively, for both the BSO and saline injection. The experiment included normoxia groups as well, with animals (control, BSO, and saline) being kept under normoxia for a total of 96 h. The anoxia groups (BSO and saline) consisted of animals exposed to 5.5 h of anoxia. The reoxygenation groups (BSO and saline) comprised animals exposed to 5.5 h of anoxia followed by reoxygenation for 15–120 min.

At their respective endpoints, the snails were euthanized by decapitation, and their hepatopancreas and foot muscle were dissected, washed in saline, blotted dry, frozen in liquid nitrogen, and stored at −80 °C.

### 2.4. Antioxidant Enzymes

The activities of catalase, glutathione transferase (GST), glutathione peroxidase (GPX), glutathione reductase (GR), and glucose 6-phosphate dehydrogenase (G6PD) were measured using spectrophotometric kinetic assays as previously described [17]. Briefly, tissue samples were homogenized in ice-cold 50 mM potassium phosphate (KPi) buffer (pH 7.2) containing 0.5 mM EDTA and 0.1 µmol/g of tissue PMSF using an ULTRA-TURRAX tissue homogenizer (IKA, Staufen, Germany). The homogenates were then centrifuged at 10,000× *g* for 15 min at 4 °C, and the resulting supernatant was collected and immediately utilized for enzymatic assays.

Catalase activity was determined by monitoring the consumption of hydrogen peroxide at 240 nm in a reaction medium consisting of 50 mM KPi, 0.5 mM EDTA, and 10 mM H_2_O_2_ [32]. Glutathione S-transferase (GST) activity was measured by monitoring the production of a glutathione (GSH) conjugate at 340 nm in a reaction medium comprising 50 mM KPi, 0.5 mM EDTA, 1 mM GSH, and 1 mM 1-chloro-2,4-dinitrobenzene [33]. Glutathione reductase (GR) activity was assessed by monitoring the consumption of NADPH at 340 nm in a reaction medium containing 50 mM KPi, 0.5 mM EDTA, 1 mM oxidized glutathione (GSSG), and 0.1 mM NADPH [34]. Glucose-6-phosphate dehydrogenase (G6PD) activity was determined by monitoring the production of NADPH at 340 nm in a reaction medium consisting of 50 mM KPi, 0.5 mM EDTA, 5 mM MgSO_4_, 1 mM glucose 6-phosphate, and 0.2 mM NADP^+^ [35]. Glutathione peroxidase (GPX) activity was measured by monitoring the consumption of NADPH in a coupled reaction system containing 50 mM KPi, 0.5 mM EDTA, 4 mM NaN_3_, 5 mM GSH, 0.1 U/mL GR (Baker’s yeast), 0.2 mM NADPH, and 0.073 mM H_2_O_2_ [36].

For all enzymes, one unit of enzymatic activity (U) was defined as the amount of enzyme that consumes or produces substrate or product at a rate of 1 μmol/min. Enzymatic activity was normalized to the total protein content, which was measured using the Bradford protein assay with bovine serum albumin as the reference protein for constructing the standard curves [37].

### 2.5. Oxidative Stress Markers and Glutathione

Colorimetric assays were used to quantify the levels of thiobarbituric acid reactive substances (TBARS) [38], protein carbonyl [39], and glutathione [40,41], as previously described [17]. Glutathione was measured as total glutathione (tGSH) and disulfide glutathione (GSSG), from which reduced glutathione (GSH) and GSSG/tGSH levels were derived.

Briefly, tissue samples were homogenized in 10% (*w*/*v*) trichloroacetic acid (TCA) using a Ten Broeck tissue homogenizer (PYREX^®^, Corning, NY, USA) on ice. Aliquots of the crude homogenate were used for thiobarbituric acid reactive substances (TBARS) quantification, while other aliquots were centrifuged at 10,000× *g* for 6 min at 4 °C. The resulting supernatant was collected and immediately utilized for glutathione measurement and kept on ice. The pellet was frozen and stored at −80 °C for protein carbonyl analysis.

For TBARS quantification, the crude homogenate was mixed with thiobarbituric acid (TBA) and HCl to achieve final concentrations of 0.25% (*w*/*v*) TBA, 0.17 M HCl, and 10% (*w*/*v*) TCA [38]. The reaction mixture was then incubated at 96 °C for 15 min, followed by centrifugation at 10,000× *g* for 6 min. The absorbance of the supernatant was measured at 532 nm and 600 nm and corrected for the “blank” tubes containing no TBA (only sample + HCl + TCA). An absorptivity coefficient of 156 mM^−1^ cm^−1^ was used to calculate TBARS levels.

Protein carbonyl levels were measured by reacting protein pellets with 10 mM 2,4-dinitrophenylhydrazine (DNPH) in 0.5 M HCl for 1 h at room temperature with vigorous vortex agitation every 15 min. The protein samples were then washed three times with 1:1 (*v*/*v*) ethanol:ethyl acetate and resolubilized with 6 M guanidine chloride in 20 mM KPi (pH 2.3). After centrifugation at 10,000× *g* for 6 min at 4 °C to remove any insoluble material, the solubilized protein samples were read at 370 nm (protein carbonyl) and 280 nm (total protein content). An absorptivity coefficient of 22,000 M^−1^ cm^−1^ was used to calculate protein carbonyl levels [39].

Glutathione levels were measured using the enzymatic recycling method. For total glutathione (tGSH), a standard curve ranging from 0.375 µM to 1.5 µM GSH was constructed using a reaction medium containing 100 mM KPi (pH 7.0), 1 mM EDTA, 0.1 mM NADPH, 0.1 mM DTNB, and 0.05 U/mL GR. For the disulfide form (GSSG), a standard curve ranging from 0.025 µM to 0.2 µM GSH was constructed using a reaction medium containing 100 mM KPi (pH 7.0), 1 mM EDTA, 0.1 mM NADPH, 0.1 mM DTNB, and 0.3 U/mL GR. The rate of increase in absorbance at 412 nm was plotted against the concentration of GSH to construct the calibration curves. For each sample, one aliquot of the supernatant was directly used to measure tGSH, while another aliquot was incubated with 2-vinylpyridine before the measurement of GSSG [42].

### 2.6. Statistics

Data normality was assessed using the Shapiro-Wilk test. The effect of BSO injection was evaluated using Kruskal-Wallis tests, comparing the following groups: control, normoxia-saline, normoxia-BSO, anoxia-saline, and anoxia-BSO (presented as column graphs). Subsequently, two-way ANOVA tests were conducted, considering oxygen and BSO as sources of variation, followed by Tukey’s multiple comparisons test (represented as point graphs). Statistical analyses and figure production were performed using GraphPad Prism 9 version 9.5.1 (GraphPad Software, San Diego, CA, USA).

## 3. Results

**Figure 1:**
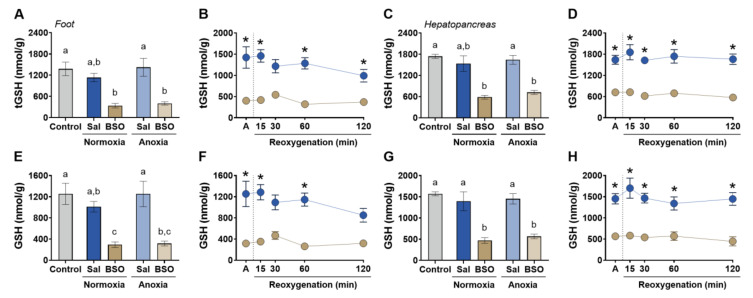
Glutathione levels in foot muscle and hepatopancreas of snails *Helix aspersa*, injected with saline or buthionine sulfoximine and maintained in normoxia or exposed to anoxia and reoxygenation. Control animals were maintained in normoxia and not injected with any substance. (**A**,**B**) Total glutathione (tGSH = GSH + 2GSSG) concentration in foot muscle (*n* = 4–7). (**C**,**D**) Total glutathione (tGSH = GSH + 2GSSG) concentration in hepatopancreas (*n* = 4–8). (**E**,**F**) Reduced glutathione (GSH) concentration in foot muscle (*n* = 4–7). (**G**,**H**) Reduced glutathione (GSH) concentration in hepatopancreas (*n* = 4–8). Groups that do not share letters are significantly different from each other (*p* < 0.05). The asterisk (*) denotes significant differences in relation to the respective BSO group (*p* < 0.05).

BSO injection significantly reduced total glutathione (tGSH) levels in both the hepatopancreas and foot muscle (Figure 1). In the foot muscle, tGSH content decreased by 75% in BSO-injected snails under normoxia and by 71% in BSO-injected snails exposed to anoxia (Figure 1A). Similarly, in the hepatopancreas, tGSH content decreased by 66% in BSO-injected snails under normoxia and by 59% in BSO-injected snails exposed to anoxia compared to the control group (Figure 1C). In both tissues, tGSH levels remained lower in BSO-injected snails than in saline-injected snails (Figure 1B,D). Similar trends were observed for reduced glutathione (GSH). GSH levels in the foot muscle decreased by 76% in BSO-injected snails under normoxia and by 75% in BSO-injected snails exposed to anoxia compared to the control group (Figure 1E). In the hepatopancreas, GSH concentration decreased by 70% in BSO-injected snails under normoxia and by 64% in BSO-injected snails exposed to anoxia compared to the control group (Figure 1G). Reduced glutathione levels in BSO-treated snails remained lower than those in saline-treated snails during reoxygenation in both the foot muscle and hepatopancreas (Figure 1F,H). These results demonstrate the effective depletion of glutathione by BSO treatment and indicate that anoxia alone did not affect tGSH or GSH levels.

**Figure 2:**
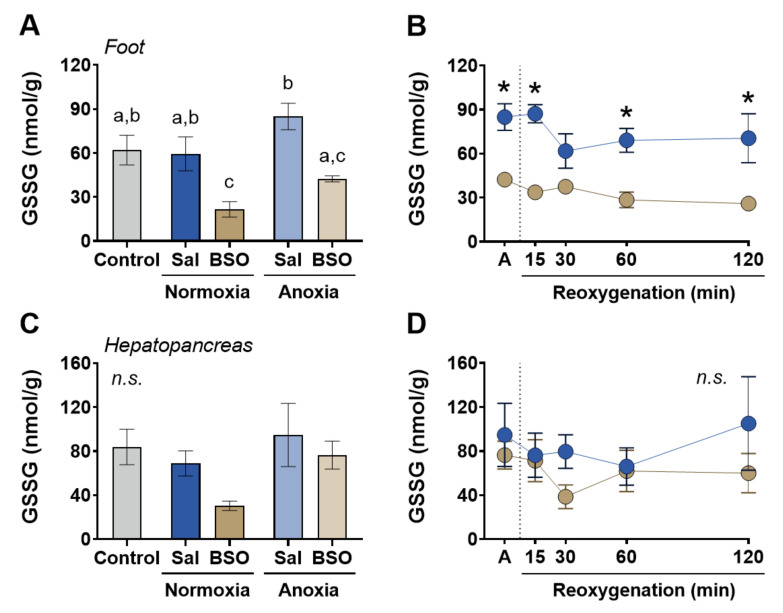
Disulfide glutathione (GSSG) levels in foot muscle and hepatopancreas of snails *Helix aspersa*, injected with saline or buthionine sulfoximine and maintained in normoxia or exposed to anoxia and reoxygenation. Control animals were maintained in normoxia and not injected with any substance. (**A**,**B**) Disulfide glutathione (GSSG) levels in foot muscle (*n* = 4–7). (**C**,**D**) Disulfide glutathione (GSSG) levels in hepatopancreas (*n* = 4–8). Groups that do not share letters are significantly different from each other (*p* < 0.05). The asterisk (*) denotes significant differences in relation to the respective BSO group (*p* < 0.05).

In the foot muscle, the levels of disulfide glutathione (GSSG) were lower in BSO-treated snails compared to saline-treated snails in both the normoxia and anoxia groups (Figure 2A). During reoxygenation, GSSG levels in the foot muscle of BSO-injected snails were lower than those in saline-injected snails (Figure 2B). In the hepatopancreas, however, there were no significant differences between BSO-injected snails and saline-injected snails in GSSG levels, although there was a trend of BSO decreasing hepatic GSSG levels in snails maintained in normoxia (Figure 2C). Anoxia and subsequent reoxygenation did not affect hepatic GSSG levels (Figure 2D). These results indicate that the levels of GSSG generally followed the pattern observed for tGSH and GSH.

**Figure 3:**
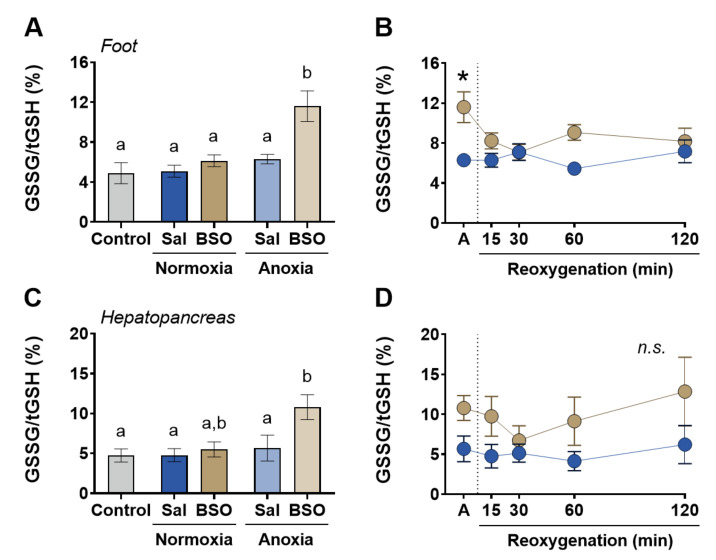
Redox balance in foot muscle and hepatopancreas of snails *Helix aspersa*, injected with saline or buthionine sulfoximine and maintained in normoxia or exposed to anoxia and reoxygenation. Control animals were maintained in normoxia and not injected with any substance. (**A**,**B**) Ratio between disulfide glutathione (GSSG) and total glutathione (tGSH = GSH + 2GSSG) in foot muscle (*n* = 4–7). (**C**,**D**) Ratio between disulfide glutathione (GSSG) and total glutathione (tGSH = GSH + 2GSSG) in hepatopancreas (*n* = 4–8). Groups that do not share letters are significantly different from each other (*p* < 0.05). The asterisk (*) denotes significant differences in relation to the respective BSO group (*p* < 0.05).

Redox balance, as indicated by GSSG/tGSH levels, remained unaffected by anoxia alone in both hepatopancreas and foot muscle. There were no significant differences observed between the control, saline-normoxia, and saline-anoxia groups (Figure 3A,C). Similarly, glutathione depletion caused by BSO alone did not alter GSSG/tGSH levels in either tissue (Figure 3A,C). However, when BSO-treated snails were exposed to anoxia, redox imbalance occurred in both tissues. GSSG/tGSH levels nearly doubled, increasing by 84% in foot muscle (Figure 3A) and by 90% in hepatopancreas (Figure 3C) compared to saline-injected snails under anoxia. During reoxygenation, GSSG/tGSH levels returned to baseline in both tissues (Figure 3B,D). Furthermore, GSSG/tGSH levels in the BSO-anoxia groups were higher than those in the control group. These findings suggest that neither glutathione depletion nor anoxia alone induces significant redox imbalance. It is only when both factors occur simultaneously that GSSG/tGSH shifts towards a more oxidized state.

There were no significant changes in the levels of protein carbonyl and thiobarbituric acid reactive substances (TBARS) during anoxia or reoxygenation, regardless of saline or BSO treatment (Table 1). These results suggest that the disturbance in redox balance did not reach a level of severity that would cause excessive oxidative damage to lipids and proteins.

**Figure 4:**
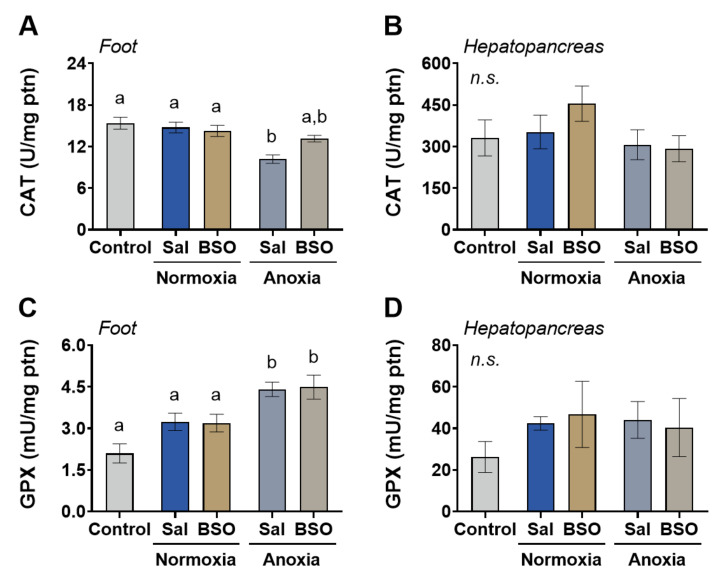
Activity of peroxide-detoxifying enzymes in foot muscle and hepatopancreas of snails *Helix aspersa*, injected with saline or buthionine sulfoximine and maintained in normoxia or exposed to anoxia. Control animals were maintained in normoxia and not injected with any substance. (**A**) Catalase activity in foot muscle (*n* = 5–7). (**B**) Catalase activity in hepatopancreas (*n* = 6–8). (**C**) Glutathione peroxidase (GPX) activity in foot muscle (*n* = 5–7). (**D**) Glutathione peroxidase (GPX) activity in hepatopancreas (*n* = 5–8). Groups that do not share letters are significantly different from each other (*p* < 0.05).

Regarding antioxidant enzyme activities, catalase activity slightly decreased in the foot muscle of saline-injected snails under normoxia (Figure 4A) but not significantly in hepatopancreas (Figure 4B). Anoxia exposure led to an increase in glutathione peroxidase (GPX) activity in the foot muscle of snails, regardless of saline or BSO injection (Figure 4C). The GPX activity in both anoxia groups (saline- and BSO-injected) was elevated by approximately 110% and 114% compared to the control group (Figure 4C). In hepatopancreas, GPX activity remained unchanged (Figure 4D).

The activities of glutathione S-transferase (GST) remained stable in both tissues in response to anoxia and BSO treatment (Figure S1). Glucose-6-phosphate dehydrogenase (G6PD) also remained stable in hepatopancreas (Figure S1). In the foot muscle, G6PD activity was 31–33% lower in both saline- and BSO-injected snails exposed to anoxia compared to the other groups (Figure S1). Glutathione reductase (GR) activity was 26–29% lower in the foot muscle of saline- and BSO-injected snails under anoxia compared to the control group (Figure S1). In the hepatopancreas, BSO-injected snails under normoxia exhibited higher GR activity (83% increase) compared to the control group (Figure S1). These oscillations in enzyme activities suggest that the foot muscle is more sensitive and responsive to stimuli compared to the hepatopancreas.

## 4. Discussion

Glutathione has a significant role in maintaining redox homeostasis in animals experiencing hypoxia/anoxia, aerial exposure (in aquatic species), and estivation and during their subsequent recovery. Indeed, glutathione levels have been shown to be upregulated in diverse animal species, from tardigrades [43] to lizards [44], exposed to environmental stresses [2]. For example, *Littorina littorea* exhibited elevated GSH levels in the muscle and hepatopancreas during anoxia exposure [45]. Similarly, mussels *Brachidontes solisianus* showed a 60% increase in whole-body GSH levels after 4 h of aerial exposure during low tide [46]. These findings indicate an enhanced antioxidant defense system to counteract redox imbalances during anoxia (in the case of *L. littorea*) or physiological hypoxia (in the case of *B. solisianus*). These biochemical responses serve as characteristic indicators of the presence of preparation for oxidative stress (POS), wherein other antioxidant defenses, including enzymes, may or may not be involved.

In our previous studies on *Helix aspersa*, we observed an increase in GSH levels during estivation, which would help regulate redox imbalance during the cycles of estivation and arousal [47,48]. The POS phenotype during estivation also involves the participation of antioxidant enzymes such as GPX and SOD. Our previous work also revealed an increase in muscle GPX levels during anoxia exposure in saline-injected *H. aspersa* [17], and this finding was replicated in the current study (Figure 4C). Most other antioxidant enzymes remained largely unchanged during anoxia in saline-injected snails. Although GSH levels did not show significant alterations during anoxia, it still plays a crucial role in the overall POS phenotype. This is because the observed increase in muscle GPX activity is effective in vivo only if GSH levels, which serve as a GPX substrate, are maintained at adequate levels. Therefore, we further investigated how the pharmacological depletion of GSH could impact the redox metabolism in snails during anoxia and reoxygenation.

Despite the significant depletion of glutathione (GSH) in BSO-injected animals, only minor changes were observed in antioxidant enzymes compared to controls or saline-injected snails. The increase in muscle GPX activity observed in saline-injected animals was also present in BSO-injected snails. Additionally, markers of oxidative stress such as TBARS and carbonyl protein remained unchanged during anoxia and reoxygenation in both saline- and BSO-injected snails. Although lipid and protein oxidation mediated by reactive oxygen and nitrogen species (RONS) appeared unaltered in GSH-depleted snails, the ratio of oxidized GSSG to total GSH (GSSG/tGSH) significantly increased during anoxia in BSO-injected snails in both tissues. However, this increased GSSG/tGSH ratio was reversed within 15–30 min of reoxygenation. These observations indicate a severe redox imbalance during anoxia in GSH-depleted animals. The decrease in GSH levels caused by BSO during anoxia led to an increased conversion of GSH into GSSG, indicating a more oxidized cellular environment proportional to the initial amount of GSH. Interestingly, GSSG levels decreased in BSO-injected normoxic or anoxic snails (reaching significance only in muscle) due to the overall depletion of glutathione induced by the drug. However, the percentage of GSSG relative to total GSH nearly doubled under anoxia in glutathione-depleted animals, providing indirect evidence of increased RONS levels and redox imbalance. It is important to note that when animals are exposed to an anoxic environment, their tissues gradually become hypoxic rather than instantly anoxic, as there is still residual oxygen present in the tissues [1]. This residual oxygen is gradually consumed over time, although not completely, and the unconsumed oxygen, being hydrophobic, is expected to be preferentially located in membranes [49,50]. The increase in the GSSG/tGSH ratio has long been recognized as a marker of disruption in redox homeostasis [51].

BSO-induced GSH depletion is associated with higher ROS levels [52,53,54,55] and activation of redox-related transcription factors [54,56]. Still, we did not observe major alterations in antioxidant systems and oxidative stress markers elicited by glutathione depletion alone in *H. aspersa*. Such a lack of antioxidant response has also been observed in other experimental systems [57,58]. The depletion of the total glutathione pool has been associated with increased tissue sensitivity to additional stresses [59,60,61,62], but not a toxic condition per se [63,64]. Studies have demonstrated that the pharmacological depletion of glutathione (GSH) exacerbates hypoxic/ischemic stress [65,66]. One notable study reported a significant increase in the GSSG/tGSH ratio in the muscle of mice exposed to 24 h of hypobaric hypoxia, which is equivalent to an altitude of 7000 m [67]. This increase in the GSSG/tGSH ratio was twice as large in glutathione-depleted mice caused by BSO injection compared to saline-injected mice. While hypoxia alone induced an increase in muscle GSSG levels, the levels of GSSG were lower in hypoxic BSO-injected mice than in saline-injected animals. These results indicate that hypoxia alone induces redox imbalance and oxidative stress (evidenced by increased TBARS levels) and that the combination of hypoxia and GSH depletion imposes even greater stress on the muscle, as indicated by the higher GSSG/tGSH ratio [67]. This redox imbalance was attributed to a higher rate of GSH oxidation to GSSG due to oxidative stress, as well as altered glutathione exchange between the muscle and circulation in response to hypoxic stress.

In snails *H. aspersa*, the increase in muscle GPX activity may have prevented oxidative stress (indicated by TBARS and carbonyl levels) in that organ in both saline- and BSO-injected anoxic animals. In the case of the hepatopancreas, the high constitutive levels of catalase may have provided protection. This is supported by the observation that catalase-depleted snails injected with ATZ exhibited increased carbonyl levels during reoxygenation [17]. However, these mechanisms were unable to prevent the redox imbalance in GSH-depleted anoxic snails. It is worth noting that redox imbalance occurs before the onset of uncontrolled oxidative stress and oxidative damage to cellular components [68,69]. It is possible that if GSH levels had been further diminished (more than 70–75% depletion) by employing higher levels of BSO, the redox scenario could have shifted towards actual oxidative stress. This hypothesis should be explored in future experiments.

At first glance, a shift towards a more oxidized state in a condition of low oxygen availability might seem counterintuitive. Initially, it was believed that exposure to hypoxia would lead to a decrease in the formation of reactive oxygen and nitrogen species (RONS) due to limited oxygen availability [7,70]. However, accumulating evidence from various animal species and cell biology experiments has shown an increase in the GSSG/tGSH ratio and oxidative damage to lipids, proteins, and DNA under hypoxic and/or anoxic conditions [13]. These findings, along with measurements of RONS using fluorescent probes, suggest increased RONS formation under hypoxia, with the mitochondrial respiratory chain being a major source of reactive species [71,72,73,74]. These pieces of evidence support the molecular basis of the POS mechanism, which involves increased RONS formation under anoxia/hypoxia or hypoxic-like conditions, followed by the activation of redox-sensitive transcription factors and subsequent increase in antioxidant defenses [7,13]. The results from anoxia-exposed snails in the present study suggest that BSO-treated animals have higher intracellular levels of RONS due to the diminished GSH pool. However, the steady-state levels of reactive species are not sufficiently high to cause oxidative damage to lipids and proteins during oxygen depletion and reoxygenation in snails.

Although the present study investigated several classic antioxidant systems, other antioxidants that were not analyzed could play a role in *H. aspersa* resistance to oxidative stress. Examples of such antioxidants include uric acid, ascorbic acid, ovothiols, and other thiol-containing molecules [75,76]. The presence of significant amounts of these molecules could potentially explain how *H. aspersa* maintains redox homeostasis without experiencing oxidative damage to proteins and lipids under anoxia/reoxygenation stress, even when GSH is depleted by 60–70%. These non-enzymatic antioxidants could compensate for such pro-oxidizing conditions. Uric acid has been associated with the POS strategy in tissues of snails *Pomacea canaliculata* during estivation [77,78] and hibernation [78,79]. Additionally, levels of ascorbic acid increase in *Pila globosa* snails during estivation [80]. These findings suggest a potential role of ascorbate and uric acid in anoxia/hypoxia tolerance in gastropods. Moreover, although ovothiols have not yet been determined in land snails, they are present in high concentrations in the organs of mussels [81] and other aquatic mollusks [82], indicating their potential relevance for redox control under anoxia/hypoxia stress. Therefore, evidence from other experimental systems suggests that these antioxidant molecules and their potential role in the POS mechanism should be investigated in *H. aspersa* and other animal models in further research.

## 5. Conclusions

In conclusion, our study demonstrates that the pharmacological inhibition of glutathione synthesis does not lead to redox imbalance or oxidative stress in land snails *H. aspersa*. Anoxia exposure and reoxygenation did not induce oxidative stress, but anoxia did trigger an antioxidant response in the muscle of these snails, characterized by increased GPX activity. However, glutathione depletion rendered the snails more susceptible to the oxidative challenge posed by oxygen depletion, resulting in a disturbance of redox homeostasis, as indicated by an elevated GSSG/tGSH ratio in both tissues of snails exposed to anoxia after BSO treatment. Importantly, our findings highlight the robust antioxidant systems present in snails *H. aspersa*, enabling them to withstand low oxygen levels and reoxygenation, as well as glutathione depletion, without experiencing significant oxidative stress. Even when exposed to both anoxia and GSH depletion, there is no evidence of oxidative damage to lipids and proteins; instead, only a shift in glutathione redox balance towards a more oxidized cellular state is observed. This remarkable ability to maintain redox homeostasis under potentially pro-oxidant conditions likely contributes to the anoxia/hypoxia tolerance observed in *H. aspersa*. These observations provide further insights into the mechanism of the POS phenomenon in invertebrates experiencing low oxygenation.

## Figures and Tables

**Table 1:** Oxidative stress markers (TBARS and protein carbonyl) in foot muscle and hepatopancreas of snails *Helix aspersa*, previously injected with saline or buthionine sulfoximine, exposed to anoxia and reoxygenation. Control animals were maintained in normoxia and not injected with any substance.

Group	Foot Muscle				Hepatopancreas			
	TBARS (nmol/gww)		Carbonyl (nmol/mg prot.)		TBARS (nmol/gww)		Carbonyl (nmol/mg prot.)	
Control	14.85 ± 2.73	5	10.41 ± 1.51	5	23.27 ± 9.41	8	11.98 ± 5.04	8
Normoxia								
Saline	16.27 ± 2.98	6	12.24 ± 2.53	5	20.13 ± 5.09	6	15.40 ± 8.64	6
BSO	15.06 ± 3.64	6	11.80 ± 1.41	6	19.87 ± 7.05	6	11.72 ± 4.07	6
Anoxia								
Saline	17.82 ± 2.94	6	9.65 ± 5.76	6	20.58 ± 6.92	8	10.78 ± 1.47	8
BSO	22.09 ± 12.37	7	10.09 ± 2.77	7	24.23 ± 6.00	7	12.97 ± 8.62	7
Reoxygenation (15 min)								
Saline	18.57 ± 3.50	7	9.82 ± 2.45	7	20.77 ± 6.45	7	10.19 ± 4.66	7
BSO	18.24 ± 5.67	7	11.16 ± 2.47	7	25.08 ± 3.71	5	8.32 ± 3.33	5
Reoxygenation (30 min)								
Saline	17.18 ± 2.91	6	10.86 ± 1.59	6	20.64 ± 11.21	6	9.87 ± 3.44	7
BSO	16.35 ± 2.19	4	8.35 ± 2.44	4	24.00 ± 10.23	5	9.26 ± 2.64	5
Reoxygenation (60 min)								
Saline	17.80 ± 4.40	7	12.08 ± 5.31	6	20.69 ± 7.17	5	8.23 ± 2.88	6
BSO	16.43 ± 2.80	7	11.58 ± 3.20	7	20.29 ± 10.69	4	9.29 ± 1.69	4
Reoxygenation (120 min)								
Saline	16.00 ± 2.74	5	11.48 ± 3.44	5	25.77 ± 6.48	5	8.24 ± 1.16	5
BSO	17.58 ± 3.66	7	10.35 ± 1.82	7	26.10 ± 12.39	7	10.33 ± 3.23	7

Data are shown as mean ± standard deviation and *n*.

## Data Availability

Data are available within the article or its Supplementary Materials.

## Supplementary Materials

The following supporting information can be downloaded at https://www.mdpi.com/article/10.3390/antiox12061197/s1: Figure S1: Enzymatic activity in foot muscle and hepatopancreas of *Helix aspersa* snails, previously injected with saline or buthionine sulfoximine, exposed to anoxia and reoxygenation.
