# Supplementary material for: Glutathione Depletion Disrupts Redox Homeostasis in an Anoxia-Tolerant Invertebrate
Source: Antioxidants (Basel). 2023 May 31;12(6):1197. doi: 10.3390/antiox12061197 (PMC10294987; doi:10.3390/antiox12061197)
Supplement: Supplementary file 1 [file antioxidants-12-01197-s001.zip › antioxidants-2386553-supplementary.pdf]

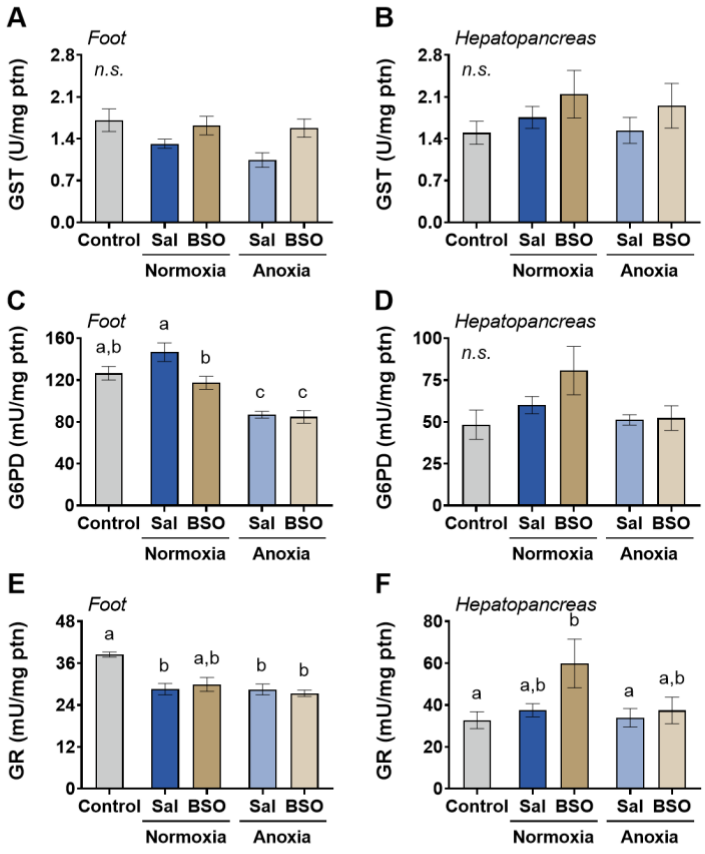

Figure S1. Activity of accessory enzymes of antioxidant systems in foot muscle and hepatopancreas of *Helix aspersa* snails, injected with saline or buthionine sulfoximine and maintained in normoxia or exposed to anoxia. Control animals were maintained in normoxia and not injected with any substance. (**A**) Glutathione transferase (GST) activity in foot muscle (N = 5–7). (**B**) Glutathione transferase (GST) activity in hepatopancreas (N = 5–8). (**C**) Glucose 6-phosphate dehydrogenase (G6PD) activity in foot muscle (N = 5–7). (**D**) Glucose 6-phosphate dehydrogenase (G6PD) activity in hepatopancreas (N = 6–8). (**E**) Glutathione redutase (GR) activity in foot muscle (N = 5–7). (**F**) Glutathione redutase (GR) activity in hepatopancreas (N = 6–8). Groups that do not share letters are significantly different from each other ($p < 0.05$).
